# The mediating role of family adaptability in the relationship between insecure attachment and family functioning in patients with major depression disorder: a cross-sectional study

**DOI:** 10.3389/fpsyt.2025.1611258

**Published:** 2025-08-20

**Authors:** Xinlei Cheng, Xinfei Li, Jinlong Zhao, Lei Wang, Xiaoning Shi

**Affiliations:** ^1^ Beijing Key Laboratory of Mental Disorders, National Clinical Research Center for Mental Disorders & National Center for Mental Disorders, Beijing Anding Hospital, Capital Medical University, Beijing, China; ^2^ Advanced Innovation Center for Human Brain Protection, Capital Medical University, Beijing, China; ^3^ Institute of Developmental Psychology, Faculty of Psychology, Beijing Normal University, Beijing, China

**Keywords:** major depressive disorders, insecure attachment, family functioning, family adaptability, mediating

## Abstract

**Introduction:**

Depression ranks as the second leading cause of the global health burden; therefore, identification of its risk and influencing factors is essential to develop more effective multidimensional interventions. The aim of the present study was to analyze the mediating role of family adaptability (FA) on the relationship between insecure attachment (IA) and family functioning (FF) in patients with major depressive disorder (MDD), which currently remains unclear. It also aims to provide a theoretical reference for optimizing rehabilitation interventions.

**Methods:**

This cross-sectional study enrolled 62 patients with MDD who were admitted to a tertiary hospital in Beijing from March 2024 to December 2024. Participants completed the General Information Questionnaire, Family Assessment Device, Family Adaptability and Cohesion Evaluation Scale, and the Experiences in Close Relationships Relationship Structures Scale. The 17-item Hamilton Depression Rating Scale was also administered. Subsequently, association analysis was performed.

**Results:**

Notable correlations were observed among IA, FA, and FF in adults with MDD. Regression analysis established both IA and FA as reliable predictors of FF. Bootstrap-mediated analysis revealed that FA partially mediated the relationship between IA and FF, accounting for 35.47% of the total association. Further analysis demonstrated differential mediation proportions; FA mediated 39.36% of the association between anxious IA and FF and 42.61% of the association between avoidant IA and FF, with all mediation pathways meeting conventional thresholds for statistical significance.

**Conclusions:**

IA in adult patients with MDD negatively impacts FF, with both anxious and avoidant IA subtypes showing independent negative associations partially linked by FA. Rehabilitation strategies should prioritize multidimensional interventions targeting the enhancement of FA to improve FF and facilitate clinical recovery.

## 1 Introduction

Major depressive disorder (MDD) was ranked as the world’s second leading level-4 cause of disability (by years lived with disability) in the 2021 Global Burden of Disease study (1). Effective first-line treatments for MDD include pharmacotherapy and psychotherapy (2, 3). Commonly used clinical psychotherapies include cognitive behavioral therapy, dialectical behavior therapy, emotion-focused therapy, and various other approaches (4). Most psychotherapies are not tailored to an individual’s living environment even though empirical evidence suggests a correlation between the characteristics of one’s living environment and the onset and persistence of depression (5). Family functioning (FF) can predict clinical outcomes in patients with MDD (6, 7). Therefore, investigating the factors and mechanisms influencing FF in adult patients with MDD has meaningful theoretical and practical relevance.

Previous studies have demonstrated a negative correlation between depression and FF (8–10). Based on McMaster’s model, FF refers to the ability of family members to fulfill their roles, appropriately engage in and respond to emotional stimuli within the family, maintain mutual cohesion, address and resolve family issues, and communicate effectively with one another (11). FF is strongly associated with depression (12–14) and quality of family attachment (15). From the perspectives of attachment and family system theories, FF appears to be related to a secure base of attachment among family members (16).

According to Bowlby’s attachment theory (17), an individual’s attachment style develops from early childhood experiences with caregivers (18). Prototype working models formed during infancy unconsciously influence expectations, fears, defenses, and behaviors, creating interpersonal experiences that persist as stable core elements throughout life and shape attachment patterns (19). Ainsworth and Bell categorized attachment into three primary types: secure, anxious, and avoidant, with the latter two classified as insecure attachment (IA) (20). Children with insufficient caregiver responsiveness tend to develop maladaptive strategies to maintain proximity to attachment figures or for self-protection, resulting in the hyperactivation (attachment anxiety) or deactivation (attachment avoidance) of the attachment system (21). Previous studies have confirmed a negative correlation between IA and FF (22), although current research has primarily focused on child and adolescent populations with limited attention to adult groups. Several meta-analyses have demonstrated associations between IA and the development, severity, chronicity, and functional impairment of MDD (23, 24). However, the specific pathways linking IA to FF and depression recovery in adults remain unclear. Therefore, the first hypothesis proposed was:

Hypothesis 1: IA is negatively associated with FF in adult patients with MDD.

According to the Circumplex Model theory, family adaptability (FA) is the ability of a family system to alter its power structure, role relationships, and relational rules in response to new situations and developmental demands (25). FA, a central characteristic of family systems, has been established to show measurable associations with depression (26), FF (27), and IA (28), with consistent evidence demonstrating predictable relationships among FA, cohesion evaluation scale outcomes, and FF (29). Clarifying the operational pathways through which IA affects FF in adult patients with depression and determining whether FA contributes to these mechanisms represents a clinically meaningful area of investigation that has received insufficient research attention. Accordingly, the second hypothesis proposed was:

Hypothesis 2: FA is associated with the IA–FF relationship, where IA correlates with lower FA, which in turn corresponds with poorer FF.

**Figure 1:**
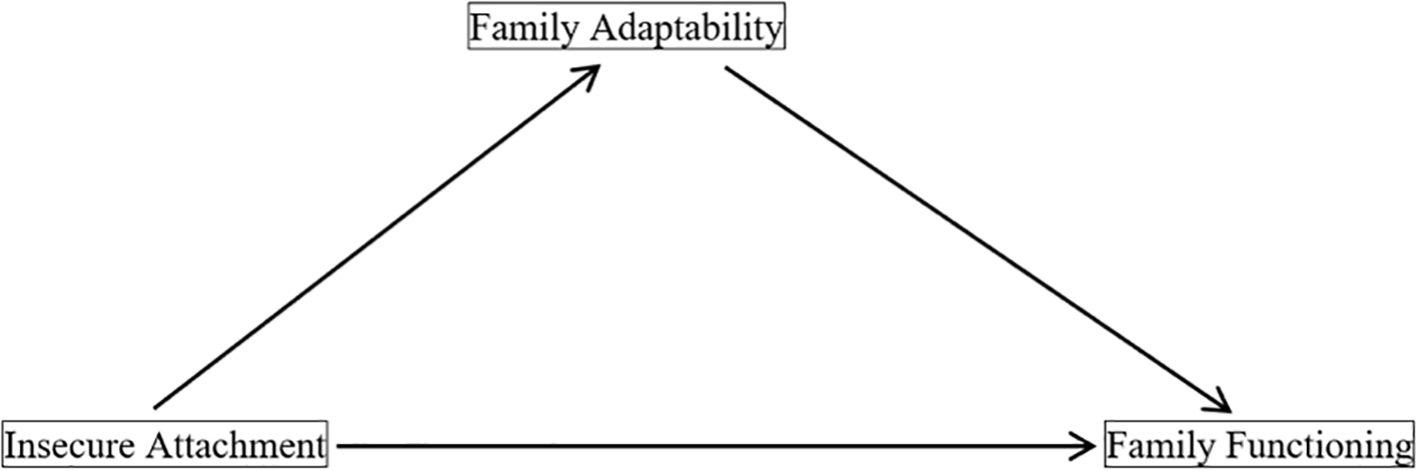
Hypothesized mediation model of family adaptability.

In summary, this study hypothesized that FF is associated with IA, and that this relationship involves FA. Consequently, we propose a mediation model (**Figure 1**) to examine the impact of IA on FF and verify the mediating role of FA, thereby providing evidence-based guidance for enhancing FF and facilitating recovery in patients with MDD.

## 2 Materials and Methods

### 2.1 Participants

From March to December 2024, inpatients at a tertiary psychiatric hospital in Beijing, China were selected as study participants. The inclusion criteria were as follows: (i) age between 18 and 60 years, (ii) diagnosis of MDD according to the International Classification of Diseases 10th Revision Classification of Mental and Behavioral Disorders (30), (iii) voluntary participation in the study, and (iv) ability to comprehend and independently complete the questionnaires. The exclusion criteria were as follows: (i) organic mental disorders, mental disorders due to psychoactive substances, bipolar affective disorder, and depressive disorder with psychotic symptoms; (ii) non-completion of the questionnaire; and (iii) substantial missing data. All the participants provided informed consent. This study was approved by the ethics committee of our hospital, (Approval No: (2023) Scientific Research No. 322). And this study was registered with the Chinese Clinical Trial Registry (registration number: ChiCTR2500101417; website: https://www.chictr.org.cn). The current manuscript introduces a sub study of this registered research project.

### 2.2 Measurements

#### 2.2.1 17-item Hamilton Depression Rating Scale

The HAMD-17 (31) is the most widely used clinical instrument for assessing depression severity. It comprises 17 items, including feelings of guilt, suicide, difficulty falling asleep, and insight. Each item is scored on a 0–2 or 0–4 scale, with the total score ranging from 0 to 54. Higher scores indicate greater depressive severity. The scoring criteria were as follows: 0–7 indicates no depression or remission period, 8–17 indicates mild depression, 18–24 indicates moderate depression, and ≥25 indicates severe depression. In this study, the scale demonstrated good reliability with a Cronbach’s α coefficient of 0.74. Owing to its dependable diagnostic accuracy and proven validity, the HAMD-17 has become widely recognized and utilized in clinical settings, as well as in research.

#### 2.2.2 Family Adaptability and Cohesion Scale, second edition

The FACES II (Chinese version) (32) is a self-administered measure that evaluates the cohesion and adaptability of families. It consists of 30 items, with each scored on a 5-point response option that ranges from 1 (“not at all”) to 5 (“always”). It includes two subscales: family cohesion (16 items) and FA (14 items). According to Olson’s theory, a higher score indicates a better FA. In this study, the scale demonstrated good reliability, with a Cronbach’s α of 0.80.

#### 2.2.3 Experiences in Close Relationships-Relationship Structures Scale

The ECR-RS (33) consists of nine items and is concise in content, comprising two dimensions—attachment avoidance (items 1–6) and attachment anxiety (items 7–9)—which align with the theoretical framework of attachment. The ECR-RS items apply to three life stages—infancy, childhood/adolescence, and adulthood–and accommodate various attachment figures. A 7-point scale from 1 (strongly disagree) to 7 (strongly agree) was used, with the first four entries reverse-scored. Higher scores indicate greater levels of attachment anxiety and avoidance. In this study, the scale demonstrated good reliability with a Cronbach’s α coefficient of 0.77.

#### 2.2.4 Family Assessment Device

The FAD (34, 35) is used to collect data on various aspects of the entire family system and can effectively identify potential problems within it. The scale consists of seven subscales: communication, roles, problem solving, affective responsiveness, affective involvement, behavioral control, and general functioning, totaling 30 items. Each item contains 4 response options (1=“Very much like my family” to 4=“Not at all like my family”). For health-positive items (Items 9–13 and 20-25), we applied reverse scoring (5 minus the actual score). All items were ultimately standardized such that 1=unhealthy and 4=healthy. Higher scores indicate a healthier FF. In this study, the scale demonstrated good reliability, with a Cronbach’s α of 0.79.

### 2.3 Data collection

Adult patients with MDD provided detailed information regarding their age, sex, marital status, family members, disease diagnosis, and depression severity. Data were collected using online questionnaires completed via mobile scanning. Researchers addressed related inquiries, and clinical information was retrieved from the patients’ medical records.

### 2.4 Statistical analysis

To address concerns regarding statistical power, we conducted a Monte Carlo power analysis for mediation using the online tool developed by Schoemann et al. (36) (https://schoemanna.shinyapps.io/mc_power_med/). We conducted 5,000 replications with a fixed sample size of 62. Based on the observed correlations among the independent variable, mediator, and dependent variable, and using a significance level of α = 0.05, the estimated power for detecting the indirect effect ranged from 0.89 to 0.99.

This study employed SPSS 23.0 statistical software to perform confirmatory factor analysis, descriptive statistics, independent samples t-tests, common method bias testing, correlation analysis, and regression analysis on normally distributed data. To assess the significance of mediation pathways, we conducted mediation model analysis using the SPSS PROCESS macro and applied the bias-corrected nonparametric percentile Bootstrap method for validation. Associations were considered statistically significant when the Bootstrap confidence intervals excluded zero.

Model 4 was used to test the mediating role of family adaptability (FA) between insecure attachment (IA) and family functioning (FF), while controlling for covariates including gender, age, education level, marital status, family composition, clinical diagnosis, and depression severity. Prior to formal analysis, we evaluated the assumptions of the linear regression model.

To further investigate the independent mediating associations of FA between anxious IA and FF, as well as between avoidant IA and FF, we re-validated the linear regression model assumptions before conducting additional analyses, followed by separate analyses using Model 4 for each relationship. To enhance the robustness of our findings, we implemented Bootstrap resampling (5,000 repetitions) to estimate coefficient distributions, assess bias and standard errors, and validate the mediation models.

## 3 Results

### 3.1 Demographics

We enrolled 62 patients with MDD (31 females and 31 males), with a mean age of 45 years. Half of the participants were married (50.0%) and almost half were mildly depressed (41.94%) (**Table 1**).

**Table 1:** Demographic, sociodemographic, and clinical characteristics of patients with major depressive disorders (*N* = 62).

Variables	*N*	%
Sex		
Male	31	50.0%
Female	31	50.0%
Age		
18–39 years	34	54.8%
40–60 years	28	45.2%
Marital status		
Single	25	40.3%
Married	31	50.0%
Divorced	6	9.7%
Family members		
Spouse	26	41.9%
Parents	31	50.0%
Children	3	4.8%
Others	2	3.2%
Diagnosis		
Depressive episodes	24	38.7%
Recurrent depressive disorder	38	61.3%
Severity of disease		
Remission period	19	30.65%
Mild	26	41.94%
Moderate	13	20.96%
Severe	4	6.45%

### 3.2 Common method bias

To address the potential common method bias, we conducted an unrotated principal component factor analysis on all scale items and performed a Harman’s single-factor test. The results revealed 21 factors with eigenvalues greater than 1, with the first factor accounting for 27.57% of the variance. This proportion was below the critical threshold of 40%, indicating the absence of significant common method bias in the study data.

### 3.3 Correlations between variables

Our analysis revealed statistically robust associations among IA, FA, and FF. The findings showed inverse relationships between IA and both FA (r = -0.512, *p* < 0.001) and FF (r = -0.593, *p* < 0.001). In contrast, a strong positive relationship was observed between the FA and FF (r = 0.659, *p* < 0.001) (**Table 2**).

**Table 2:** Correlation between insecure attachment, family adaptability, and family functioning.

Variables	*M*	*SD*	1	2	3
1. Insecure Attachment	30.08	12.35	1		
2. Family Adaptability	45.00	11.87	-0.512***	2	
3. Family Functioning	83.32	16.11	-0.593***	0.659***	3

*p < 0.05, **p < 0.01, ***p < 0.001.

After categorizing the IA subtypes, Pearson analyses were performed to examine the association between attachment and FF. Anxious IA was inversely correlated to FA (r = -0.378, *p* < 0.01) and FF (r = -0.458, *p* < 0.001), whereas avoidant IA showed a comparable negative correlation with FA (r = -0.470, *p* < 0.001) and FF (r = -0.458, *p* < 0.001). FA and FF remained positively correlated (r = 0.659, *p* < 0.001) (**Table 3**).

**Table 3:** Correlation between anxious insecure attachment, avoidant insecure attachment, family adaptability, and family functioning.

Variables	*M*	*SD*	1	2	3	4
1. Anxious Insecure Attachment	16.34	7.27	1			
2. Avoidant Insecure Attachment	13.74	7.63	0.375**	2		
3. Family Adaptability	45.00	11.87	-0.378**	-0.470***	3	
4. Family Functioning	83.32	16.11	-0.458***	-0.524***	0.659***	4

*p < 0.05, **p < 0.01, ***p < 0.001.

Model 4 examined the mediating associations of FA on IA and FF. The analysis was controlled for sex, age, marital status, family members, disease diagnosis, and severity of depression. Prior to analysis, we evaluated the assumptions of the linear regression. To further investigate whether FA accounts for the associations between anxious/avoidant IA and FF, we revalidated the assumptions of the linear regression model before conducting additional mediation analyses. The results indicated that IA was inversely associated with FA and negatively correlated with FF, while FA showed a positive association with FF (**Table 4**). Specifically, anxious and avoidant IA reduced FA and directly worsened FF, whereas FA consistently enhanced it (**Table 4**).

**Table 4:** Multiple linear regression to identify predictors of family functioning.

Outcome variable	Predictor variable	Coefficient significance			Fit indices		
		*β*	*t*	*p*	*R*	*R^2^*	*F*
Family Functioning	Family adaptability	0.591	3.326	0.002**	0.761	0.580	7.962
	IA	-0.433	-2.848	0.006**			
	Sex	-0.051	-0.015	0.988			
	Age	-6.046	-1.570	0.122			
	Marital status	5.491	1.961	0.055			
	Family members	-0.450	-0.194	0.847			
	Diagnosis	2.204	0.623	0.536			
	Severity of disease	-0.206	-0.989	0.327			
Family Functioning	Family adaptability	0.689	3.982	< 0.001***	0.746	0.556	7.244
	Anxiety IA	-0.549	-2.230	0.030*			
	Sex	-0.128	-0.037	0.970			
	Age	-7.630	-1.919	0.060			
	Marital status	5.840	2.031	0.047			
	Family members	-0.615	-0.258	0.798			
	Diagnosis	3.402	0.899	0.373			
	Severity of disease	-0.271	-1.290	0.203			
Family Functioning	Family adaptability	0.681	3.847	< 0.001***	0.743	0.551	7.101
	Avoidant IA	-0.505	-2.085	0.042*			
	Sex	0.650	0.191	0.849			
	Age	-5.017	-1.240	0.220			
	Marital status	5.319	1.836	0.072			
	Family members	-0.095	-0.039	0.969			
	Diagnosis	-0.009	-0.002	0.998			
	Severity of disease	-0.283	-1.343	0.185			

*p < 0.05; **p < 0.01; ***p < 0.001. IA, insecure attachment.

### 3.4 Mediation association analysis

**Figure 2:**
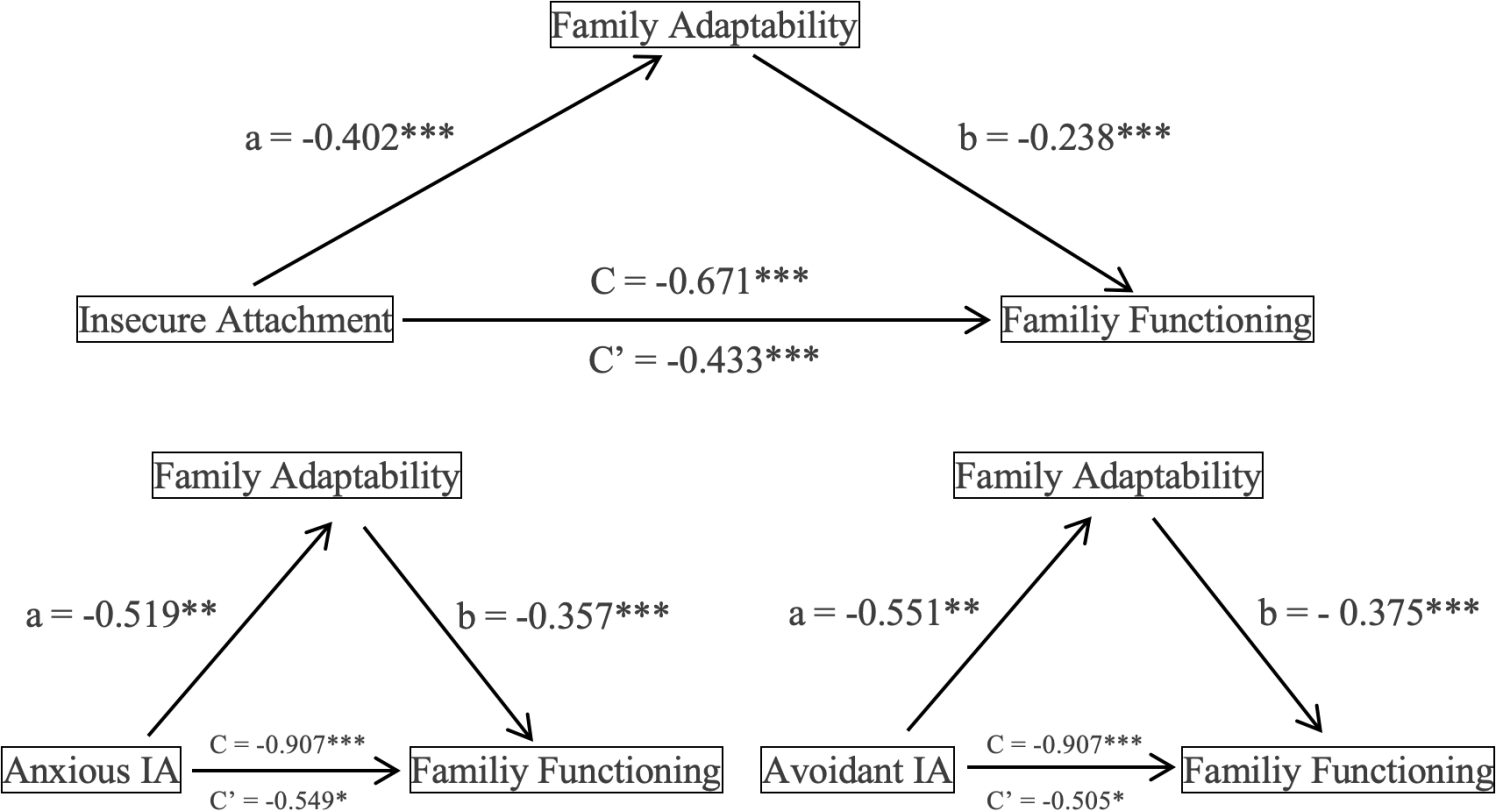
Intermediary role of family adaptability. Nonstandardized coefficients are reported. *N* = 62. *p < 0.05, **p < 0.01, ***p < 0.001. C indicates total effect; C’ indicates direct effect. IA, Insecure Attachment. Arrows only represent statistical associations,not causal direction.

Model 4 examined the mediating role of FA on IA and FF. To enhance the robustness of our findings, the bootstrap function of the boot package was applied with 5,000 resamples to estimate the coefficient distributions, assess bias and standard errors, and evaluate the mediation model. Version 4.1 of the PROCESS macro in SPSS was used for mediation analysis, with associations considered statistically significant if bootstrap confidence intervals excluded zero. To assess whether FA differentially accounts for the associations between attachment subtypes and FF, separate analyses were conducted for anxious and avoidant IA using Model 4. These results indicated that FA mediated the association between IA and FF. IA showed significant associations with FF through both direct and indirect pathways involving FA, with mediation accounting for 35.47% of the total observed association. Furthermore, FA partially mediated the relationship between the anxious and avoidant IA subtypes and FF. For both attachment patterns, the total, direct, and indirect association on FF reached the conventional thresholds for interpretation, with mediation proportions of 39.40% for anxious IA, and 42.61% for avoidant IA (**Figure 2**, **Table 5**).

**Table 5:** The intermediary role of family adaptability in the insecure attachment-family functioning association.

Model		Effect	Boot SE	Bootstrap 95%CI		Ratio of indirect to total effect
				Boot LLCI	Boot ULCI	
IA-FA-FF	Total effect	-0.671	0.146	-0.964	-0.377	
	Direct effect	-0.433	0.152	-0.738	-0.128	
	Indirect effect	-0.238	0.104	-0.463	-0.060	35.47%
Anxious IA-FA-FF	Total effect	-0.906	0.259	-1.427	-0.386	
	Direct effect	-0.549	0.246	-1.043	-0.055	
	Indirect effect	-0.357	0.158	-0.690	-0.065	39.40%
Avoidant IA-FA-FF	Total effect	-0.880	0.249	-1.380	-0.381	
	Direct effect	-0.505	0.242	-0.991	-0.019	
	Indirect effect	-0.375	0.161	-0.728	-0.097	42.61%

*p < 0.05, **p < 0.01, ***p < 0.001. IA, insecure attachment; FA, family adaptability; FF, family functioning; LLCI, lower limit confidence interval; SE, standard error; ULCI, upper limit confidence interval.

## 4 Discussion

### 4.1 Predictive role of IA on FF in adult patients with MDD

This study confirmed that IA negatively predicts FF in adult patients with MDD, which is consistent with previous research on attachment patterns and FF (22, 37). Higher IA levels are associated with greater communication barriers, more rigid family interactions, and poorer FF.

First, individuals with anxious IA tend to engage in excessive rumination during family communication and emotional engagement, persistently seek reassurance about their worth and partner availability (38), and frequently exhibit jealousy and emotional instability in close relationships (39). Their heightened vigilance and overreactions to perceived threats impair their capacity to adaptively fulfill family roles when facing new situational demands (40). Consequently, their problem-solving abilities deteriorate, which ultimately compromises FF. In contrast, individuals with avoidant IA tend to avoid attachment-related thoughts and feelings predominantly by employing deactivating emotion regulation strategies (41, 42). When facing family conflicts or problems, they more frequently choose silence or withdrawal rather than active problem solving to avoid confrontation (43). This avoidance behavior leads to failure to fulfill family responsibilities, ultimately resulting in impaired FF.

We discovered that IA negatively predicted FF, whereas FF showed an inverse relationship with MDD. Dysfunctional family environments hinder recovery from depression (44, 45), indicating that IA is not only related to depression onset, progression, and severity (45–48), but also complicates treatment outcomes. These findings suggest that future research should develop individualized family interventions tailored to different attachment patterns to reduce IA, improve FF, and facilitate long-term recovery. These results underscore the importance of interdisciplinary collaboration, particularly between psychiatry and psychology, in establishing comprehensive long-term management strategies for adult patients with chronic depressive disorder.

### 4.2 FA’s intermediary role in the IA-FF association in adult patients with MDD

This study identified FA as a critical mediator between IA and FF in adult patients with MDD, establishing a sequential pathway from IA to impaired FA, and ultimately to poorer FF. Specifically, higher levels of IA were associated with reduced FA levels, which in turn led to poorer FF. FA’s intermediary role showed a substantial association with this complex process. These findings align with those of previous studies on child and adolescent populations (28, 49), further confirming the universal negative impact of IA on both FA and FF across different age groups and clinical/non-clinical populations.

Notably, the results further revealed differential mediation patterns. FA mediated the relationship between FF and both anxious and avoidant IA, but with distinct effect magnitudes, demonstrating attachment-specific mediation mechanisms. These findings offer a refined perspective on the relationship between IA and FF in adults with MDD.

Individuals with anxious and avoidant IA demonstrate substantial differences in emotional interactions, communication patterns, and problem-solving capacities within their family systems. Research indicates that when distressed, anxiously attached individuals often amplify their suffering (attachment hyperactivation) to elicit caregiver attention, predominantly employing over-activating emotion regulation strategies characterized by catastrophizing and rumination (50). This pattern reduces FF, promotes maladaptive coping, undermines consistent emotional support and security, and ultimately impairs FF. Individuals with avoidant attachment typically distrust others’ responsiveness, maintain emotional distance from attachment figures, and reject intimacy to prevent disappointment. Their characteristic pain suppression (attachment deactivation) and predominant use of deactivating emotion regulation strategies hinder meaningful emotional exchanges with family members (51, 52). This impairment in FA manifests as difficulty in open discussions during new family challenges and the inability to adjust relational rules or role allocations, ultimately degrading FF.

Attachment patterns are established during early childhood (53) and demonstrate considerable stability in adulthood, typically requiring prolonged systematic intervention for modification (18, 19) and presenting substantial treatment challenges for depression. This study revealed the mediating role of FA in the relationship between IA and FF in adult patients with depression, identifying FA as a crucial factor in the observed relationship between IA and FF. These findings suggest that therapeutic interventions targeting FA, particularly those that improve communication patterns, enhance emotional expression, and strengthen problem-solving skills within family systems, may effectively mitigate the negative association between IA and FF. Such approaches could optimize the living environment of patients with depression and create more favorable conditions for clinical recovery.

### 4.3 Study limitations and future directions

This study investigated the impact of IA on FF and its underlying mechanisms in patients with MDD to provide a reference for the development of rehabilitation plans. However, the study design incorporated the following limitations. First, this study explored the mediating role of family adaptability between insecure attachment and family functioning in patients with depressive disorder. Although the sample size was relatively small, future studies with larger samples are warranted to validate these findings. Second, this study employed a cross-sectional design that only permitted path analysis among variables without establishing causal inferences. Future longitudinal studies with expanded sample sizes could stratify depression severity and reassess patients after family therapy interventions. Subsequent evaluations of changes in attachment styles, family adaptability, and family functioning would yield additional data for analysis. Such approaches may elucidate more pathways and mechanisms between insecure attachment and family functioning, thereby providing theoretical references for depression rehabilitation.

## 5 Conclusion

In summary, this study elucidated the impact of IA on FF and its underlying mechanisms in patients with MDD. This supports the direct negative association between IA and FF, and identifies the mediating role of FA between IA and FF. Further analysis identified distinct intermediary pathways and association patterns. That is, FA differentially mediated the relationship between anxious IA and FF versus avoidant IA and FF. Consequently, when developing rehabilitation plans for depressive disorders, clinicians should focus on improving the family environment through the active implementation of family therapy. Particular attention should be paid to tailoring treatment strategies according to attachment characteristics, enhancing FA, and improving FF to facilitate patient recovery.

## Data Availability

The raw data supporting the conclusions of this article will be made available by the authors, without undue reservation.
